# Vibegron in overactive bladder: a comprehensive review of efficacy, safety and patient-reported outcomes

**DOI:** 10.1007/s00345-025-05799-8

**Published:** 2025-08-26

**Authors:** Benoit Peyronnet, Benjamin M. Brucker, Cosimo De Nunzio, Christian Gratzke, John Heesakkers, Martin C. Michel, Maurizio Serati, David Staskin, Christopher Chapple

**Affiliations:** 1 Department of Urology, Service dUrologie, Hopital Pontchaillou, University Hospital of Rennes, 2 Rue Henri Le Guilloux, 35000 Rennes, France; 2 Department of Urology and Obstetrics & Gynaecology, NYU Langone Health, New York, NY USA; 3 Department of Urology, Ospedale Sant’Andrea, Sapienza University of Rome, Rome, Italy; 4 Department of Urology, Faculty of Medicine, University Freiburg, Freiburg Im Breisgau, Baden-Württemberg Germany; 5 Department of Urology, Maastricht UMC+, Maastricht, Netherlands; 6 Department of Pharmacology, University Medical Center, Johannes Gutenberg University, Mainz, Germany; 7 Department of Obstetrics and Gynecology, Urogynecology Unit, University of Insubria, Varese, Italy; 8 Tufts University School of Medicine, Boston, MA USA; 9 Sheffield Teaching Hospitals and The University of Sheffield, South Yorkshire, Sheffield, UK

**Keywords:** β_3_-adrenoceptor agonists, β_3_-adrenergic receptor agonists, Overactive bladder, Pharmacokinetics, Treatment adherence and compliance, Vibegron

## Abstract

**Introduction:**

Overactive bladder (OAB) is a prevalent and potentially debilitating syndrome that significantly impairs quality of life. Mirabegron and vibegron are β_3_-adrenoceptor (β_3_AR) agonists that provide a different mechanism of action to antimuscarinic medications. Vibegron has high β_3_AR selectivity and enhances detrusor relaxation without compromising voiding function. This review summarises the clinical and real-world evidence supporting the efficacy, safety and patient-reported benefits of vibegron in OAB.

**Methods:**

A comprehensive search of the PubMed database was conducted in December 2024 using the keyword "vibegron". This search yielded 123 entries, which were subsequently screened by title for relevance to the objectives of this narrative review. All relevant articles identified through this process were included.

**Results:**

Pivotal phase III trials have demonstrated significant reductions in urgency, urinary frequency and urgency urinary incontinence with vibegron, with rapid onset of action and a more favourable tolerability profile than antimuscarinics. The benefits of vibegron were consistent across diverse patient populations, including older adults and those with concomitant benign prostatic hyperplasia. Real-world data further suggest that vibegron is associated with improved adherence and persistence compared with other OAB therapies. Additionally, cardiovascular safety studies confirm that vibegron has no clinically significant effects on blood pressure or heart rate. While comparative trials with mirabegron indicate similar efficacy, vibegron’s higher β_3_AR selectivity and lack of cytochrome P450 interactions offer advantages in specific patient groups. Ongoing research, including real-world phase IV studies, aims to further define the long-term effectiveness and safety of vibegron in clinical practice.

**Conclusion:**

Vibegron represents an important advance in the pharmacologic management of OAB, providing a well-tolerated and effective alternative to existing therapies.

## Introduction

Overactive bladder (OAB) is a complex symptom syndrome defined by the International Continence Society as urgency, often accompanied by frequency, nocturia and urgency urinary incontinence (UUI) [1, 2]. OAB is prevalent, affecting an estimated 546 million individuals globally, or 10.9% of the worldwide population aged ≥ 20 years [3]. OAB has a prevalence of up to 17.4% in men and 16.0% in women [4, 5]. The syndrome is associated with significant health-related quality of life (HRQOL) impairments, economic burden and stigmatisation [3].

Antimuscarinic therapies remain a cornerstone of pharmacological treatment for OAB and are often used as first-line pharmacological options, frequently in conjunction with behavioural therapies such as pelvic floor exercises and bladder training [6–10]. However, anticholinergic therapies are associated with significant challenges, including poor long-term adherence, with over 90% of patients discontinuing treatment within 2 years, and a rapid decline in persistence within the first 6 months [11, 12]. Common adverse events (AEs) such as dry mouth and constipation limit tolerability [11]. Furthermore, long-term use is linked to cognitive impairment and dementia, particularly in older adults, with bladder antimuscarinics showing a strong association with these risks [11–13].

β_3_-adrenoceptor (β_3_AR) agonists relax detrusor smooth muscle during the bladder storage phase, and as such, represent an alternative treatment for OAB [14, 15]. The first orally active β_3_AR agonist, mirabegron, is as effective as antimuscarinic agents and is better tolerated [16]. Vibegron is a selective β_3_AR agonist, with higher affinity and lower likelihood to be metabolized by cytochrome P450 (CYP450) enzymes than mirabegron [17]. Vibegron offers a novel mechanism of action distinct from antimuscarinics, improving storage function without compromising voiding efficiency [7]. The favourable safety profile, lack of significant off-target effects, and minimal drug-drug interactions position vibegron as a promising alternative, particularly for patients who cannot tolerate or benefit from antimuscarinic therapies [7, 8].

This review summarises the clinical evidence supporting the use of vibegron in OAB, with a particular focus on efficacy, safety and patient-reported outcomes.

## Methods

A comprehensive search of the PubMed database was conducted in December 2024 using the keyword "vibegron". This search yielded 123 entries, which were subsequently screened by title for relevance to the objectives of this narrative review. All relevant articles identified through this process were included. To ensure comprehensive coverage, the reference lists of these articles were manually reviewed. Additional pertinent studies were incorporated on an ad hoc basis.

## Pathophysiology of OAB

OAB has been historically considered to be associated with detrusor overactivity. However, detrusor overactivity alone fails to explain OAB symptoms in a significant subset of patients, suggesting a more complex pathophysiology [2].

Traditionally, OAB has been understood through the myogenic hypothesis, which attributes symptoms to intrinsic dysfunctions of the detrusor muscle [2]. According to this hypothesis, aberrant electrical coupling between smooth muscle cells, possibly induced by denervation or structural alterations, synchronises micromotions into involuntary detrusor contractions. However, detrusor overactivity has been reported to be absent in approximately 36% of patients with OAB [18]. Therefore, several other pathophysiological mechanisms are now believed to contribute to OAB [2]. The urothelial and suburothelial hypothesis suggests that altered sensory signalling from the bladder lining plays a critical role in OAB [2]. According to this hypothesis, dysfunctions in the urothelium, such as aberrant neurotransmitter release or changes in suburothelial interstitial cells, enhance afferent nerve signalling and amplify sensations of urgency [2, 19]. Central neural dysregulation is also implicated in OAB, particularly in cases of urgency without demonstrable detrusor overactivity [19, 20]. Dysfunctional central inhibitory mechanisms or miscommunication between the periaqueductal grey matter and cortical centres exacerbate sensory perception, contributing to OAB symptoms [19, 20].

Emerging evidence identifies additional systemic and local contributors to OAB pathophysiology [2]. These include metabolic syndrome, hormonal imbalances and alterations in the urinary microbiome. These factors, along with subclinical autonomic dysfunction, support the notion that OAB is not a singular disorder but rather a constellation of overlapping phenotypes, each driven by unique underlying mechanisms [2]. It is likely that the various phenotypes of OAB do not respond to all OAB therapies in the same way, although there is currently not enough evidence to fully support this hypothesis.

## β_3_ adrenoceptors in bladder function

β_3_ARs mediate relaxation of the detrusor smooth muscle during the storage phase of the micturition cycle [19, 21, 22]. First identified as a distinct adrenoceptor subtype in 1989, β_3_ARs are highly expressed in the human bladder, as well as in suburothelial interstitial cells and, to a lesser extent, in the urothelium. The activation of β_3_ARs facilitates bladder filling by suppressing detrusor microcontractions and reducing acetylcholine release from parasympathetic nerves, thereby enhancing bladder compliance without impairing voiding function [19–21]. Beyond its role in motor control, β_3_AR activation influences sensory pathways in the bladder [21, 22]. Studies suggest that β_3_AR agonists modulate afferent nerve activity, particularly by attenuating mechanosensitive Aδ- and C-fibre signalling during bladder filling. This modulation underpins the therapeutic benefit of β_3_AR agonists in managing urgency and frequency, the hallmark symptoms of OAB [21, 22]. In comparative analyses, β_3_AR-mediated interventions for OAB offer significant advantages over antimuscarinic therapies [19, 21]. These include minimal interference with bladder contractility, reduced risk of urinary retention and a favourable AE profile. The focus on sensory pathway modulation rather than motor inhibition positions β_3_ARs as a promising target for contemporary OAB therapies [19, 21].

## Pharmacokinetics, pharmacodynamics and selectivity of vibegron

Vibegron is a potent and highly selective β_3_AR agonist approved for the treatment of OAB [7, 14]. It exhibits favourable oral bioavailability and rapid absorption, with a terminal half-life of 60–70 h, supporting once-daily dosing [7]. In addition, vibegron tablets are able to be crushed and mixed with certain soft foods, e.g. apple sauce, with no meaningful change in pharmacokinetic parameters, which may be important for patients who have difficulty swallowing tablets [23]. Peak plasma concentrations are reached within 1–3 h of administration, and steady-state concentrations are achieved after 7 days of daily administration. Unlike other β_3_ARs such as mirabegron, vibegron is primarily metabolised through oxidation and glucuronidation and does not significantly inhibit or induce CYP450 enzymes, including CYP3A4 or CYP2D6. This profile minimises the risk of clinically significant drug-drug interactions, making vibegron particularly suitable for patients on polypharmacy regimens [7]. A comparative analysis of prescribing information for vibegron and mirabegron is shown in Table 1.

**Table 1:** Comparative analysis of prescribing information for mirabegron and vibegron for the treatment of overactive bladder [47–50]

	Vibegron		Mirabegron		
	Europe	USA	Europe		USA
Dosing and administration					
General	Fixed: 75 mg May crush, mix with certain soft foods, e.g. apple sauce		Fixed: 50 mg Do not chew, divide or crush		Titration: Start at 25 mg, ↑ to 50 mg according to efficacy/tolerability Do not chew, divide, or crush
Renal impairment	Mild to severe: no adjustment ESRD: not recommended*		Mild to moderate + strong CYP3A inhibitors: do not exceed 25 mg Severe: do not exceed 25 mg Severe + strong CYP3A inhibitors: not recommended ESRD: not recommended*		Mild to moderate: 25–50 mg Severe: do not exceed 25 mg ESRD: not recommended*
Hepatic impairment	Mild to moderate: no adjustment Severe: not recommended*		Mild + strong CYP3A inhibitors: do not exceed 25 mg Moderate: do not exceed 25 mg Moderate + strong CYP3A inhibitors: not recommended Severe: not recommended*		Mild: 25–50 mg Moderate: do not exceed 25 mg Severe: not recommended*
Warnings/precautions					
Drug interactions	Does not inhibit or induce CYP450 enzymes If co-administered with digoxin, monitor serum digoxin concentrations to titrate digoxin dose to desired clinical effect	Does not inhibit or induce CYP450 enzymes Measure serum digoxin concentrations before initiating vibegron Monitor serum digoxin concentrations to titrate digoxin dose to desired clinical effect	Use with appropriate monitoring and possible dose adjustment of drugs metabolised by CYP2D6 If co-administered with digoxin, use the lowest dose of digoxin Monitor serum digoxin concentrations to titrate digoxin dose to desired clinical effect		
Hypertension	No warnings/precautions		Periodic BP monitoring recommended Severe uncontrolled: contraindicated	Periodic BP monitoring recommended Severe uncontrolled: not recommended	
QT prolongation	No warnings/precautions		Caution in patients with known QT interval prolongation	No warnings/precautions	
Urinary retention	Monitor patients with BOO and those taking antimuscarinics for OAB, for signs and symptoms of urinary retention		Caution in patients with BOO and in those taking antimuscarinics for OAB because of the risk of urinary retention		
Angioedema	No warnings/precautions		Reported on face, tongue and/or throat		Reported on face, lips, tongue, and/or larynx

*BOO* bladder outlet obstruction, *BP* blood pressure, *CYP* cytochrome P, *ESRD* end-stage renal disease, *OAB* overactive bladder

^*^Not studied in this population

**Fig. 1:**
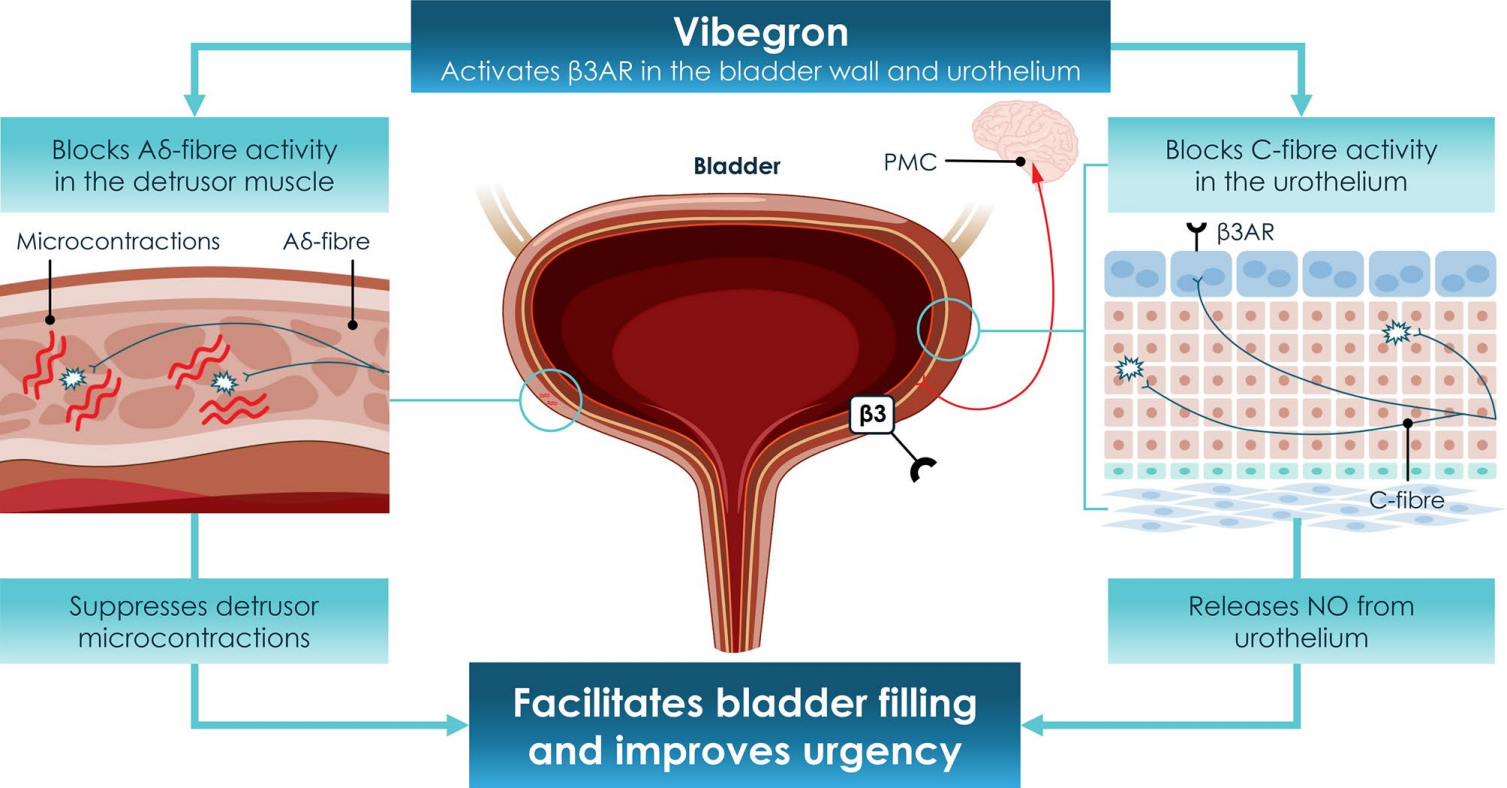
Location of β_3_ARs in the detrusor muscle of the bladder wall and mechanism of action of vibegron. Vibegron activates β_3_ARs to relax the detrusor muscle to increase bladder capacity. *β*_*3*_*AR* β_3_-adrenoceptor, *NO* nitric oxide, *PMC* pontine micturition centre

Preclinical studies demonstrated that vibegron does not penetrate into the central nervous system (CNS), as it does not cross the blood–brain barrier [7, 24]. This property significantly reduces the likelihood of CNS-related AEs, such as cognitive impairment, which are often associated with anticholinergic therapies. This aspect makes vibegron a promising option for elderly patients or those with conditions such as Alzheimer’s disease, where preservation of cognitive function is critical [7]. Vibegron enhances bladder compliance by selectively activating β_3_ARs (Fig. 1), increasing cyclic AMP production and stimulating downstream signalling pathways. This action facilitates detrusor muscle relaxation during the bladder’s filling phase, improving symptoms such as urgency, frequency and UUI without compromising voiding efficiency [7, 14]. Unlike antimuscarinic agents, which act via cholinergic pathways, vibegron achieves bladder relaxation without affecting detrusor motor function, providing an alternative for patients intolerant to traditional therapies [7].

Selectivity is a key attribute of vibegron, with β_3_AR selectivity > 7937- to > 9000-fold over β_1_ARs and β_2_ARs under standardised experimental conditions [25, 26]. In vitro studies demonstrated that vibegron exhibits minimal activity at β_1_ARs or β_2_ARs (EC_50_ > 10 µM) [25, 26], confirming its superior β_3_AR selectivity compared with other agents, such as mirabegron, which shows β_3_AR selectivity of 517- and 496-fold over β_1_ARs and β_2_ARs, respectively [25]. This high degree of selectivity minimises cardiovascular off-target effects, such as tachycardia or hypertension, which are more common with less selective agents [25]. Additionally, vibegron maintains full agonist activity at β_3_ARs regardless of receptor density, a unique feature that further supports its therapeutic efficacy and safety in OAB treatment [25].

## Clinical studies of vibegron

### Phase II studies

Vibegron has been extensively evaluated in clinical studies [27]. A phase IIb trial supported the efficacy and safety of vibegron [28]. This randomised, double-blind study included an active control (tolterodine) and evaluated a range of vibegron doses in patients with OAB. The trial reported significant reductions in urinary frequency, urgency episodes and UUI episodes with vibegron 50 mg and 100 mg compared with placebo (p < 0.05), as well as improvements in HRQOL measures (Table 2). Efficacy was sustained throughout a 52-week extension study [29], underscoring the long-term benefits of vibegron. These trials provided critical insights into the optimal dosing and safety profile of vibegron 50 mg and 100 mg, paving the way for its phase III development.

**Table 2:** Efficacy outcomes with vibegron in significant clinical trials and subgroup analyses

Study, reference	Design follow-up duration, weeks	Population	Treatment	CFB in micturitions/day, LSM (95% CI)	CFB in UUI episodes/day, LSM (95% CI)	CFB in urgency episodes/day, LSM (95% CI)	CFB in total urinary incontinence episodes, LSM (95% CI)
Yoshida et al. 2018 [30]	Phase III, R, DB, PC, AC 12 weeks	≥ 18 years old, history of OAB, ≥ 8.0 micturitions/day	Vibegron 50 mg OD (n = 370)	‒ 2.08 (‒ 2.27 to ‒ 1.89)*	‒ 1.35 (‒ 1.48 to ‒ 1.23)**	‒ 2.28 (‒ 2.46 to ‒ 2.09)*	‒ 1.4 (‒ 1.54 to ‒ 1.26)**
			Vibegron 100 mg OD (n = 368)	‒ 2.03 (‒ 2.22 to ‒ 1.84)*	‒ 1.47 (‒ 1.60 to ‒ 1.34)*	‒ 2.44 (‒ 2.63 to ‒ 2.25)*	‒ 1.53 (‒ 1.67 to ‒ 1.38)*
			Imidafenacin 0.1 mg BID (n = 117)	‒ 2.06 (‒ 2.39 to ‒ 1.73)	‒ 1.51 (‒ 1.73 to ‒ 1.29)	‒ 2.15 (‒ 2.47 to ‒ 1.82)	‒ 1.47 (‒ 1.71 to ‒ 1.23)
			Placebo (n = 369)	‒ 1.21 (‒ 1.40 to ‒ 1.03)	‒ 1.08 (‒ 1.21 to ‒ 0.96)	‒ 1.77 (‒ 1.96 to ‒ 1.58)	‒ 1.1 (‒ 1.24 to ‒ 0.96)
Yoshida et al. 2018 [31]	Phase III, OL, NC 52 weeks	≥ 20 years old, history of OAB, ≥ 8.0 micturitions/day	Vibegron 50 mg OD, dose retained (n = 118)	‒ 2.71 (‒ 3.21 to ‒ 2.21)	‒ 1.55 (‒ 1.88 to ‒ 1.22)	‒ 2.91 (‒ 3.43 to ‒ 2.39)	‒ 1.61 (‒ 1.97 to ‒ 1.26)
			Vibegron 100 mg OD, dose escalated (n = 51)	‒ 3.16 (‒ 3.90 to ‒ 2.41)	‒ 2.29 (‒ 2.76 to ‒ 1.81)	‒ 3.42 (‒ 4.20 to ‒ 2.65)	‒ 2.39 (‒ 2.90 to ‒ 1.89)
Mitcheson et al. 2019 [28]	Phase IIb, R, DB, PC, AC 8 weeks	18–75 years old, history of OAB, ≥ 8.0 micturitions/day	Vibegron 3 mg OD (n = 144)	‒1.56 (‒1.97 to ‒ 1.15)	‒ 1.38 (‒ 1.73 to ‒ 1.03)	‒ 1.69 (‒ 2.12 to ‒ 1.26)	‒ 1.56 (‒ 1.97 to ‒ 1.15)
			Vibegron 15 mg OD (n = 134)	‒ 1.71 (‒ 2.22 to ‒ 1.20)	‒ 1.81 (‒ 2.25 to ‒ 1.37)**	‒ 2.35 (‒ 2.85 to ‒ 1.85)	‒ 1.99 (‒ 2.50 to ‒ 1.48)***
			Vibegron 50 mg OD (n = 150)	‒ 1.87 (‒ 2.25 to ‒ 1.49)**	‒ 1.9 (‒ 2.35 to ‒ 1.45)*	‒ 2.36 (‒ 2.82 to ‒ 1.90)***	‒ 2.02 (‒ 2.48 to ‒ 1.56)**
			Vibegron 100 mg OD (n = 261)	‒ 2.11 (‒ 2.49 to ‒ 1.73)*	‒ 2.05 (‒ 2.50 to ‒ 1.60)*	‒ 2.98 (‒ 3.46 to ‒ 2.50)*	‒ 2.26 (‒ 2.75 to ‒ 1.77)**
			Tolterodine ER 4 mg OD (n = 257)	‒ 1.73 (‒ 2.11 to ‒ 1.35)***	‒ 1.67 (‒ 2.10 to ‒ 1.24)***	‒ 2.52 (‒ 3.00 to ‒ 2.04)**	‒ 1.8 (‒ 2.27 to ‒ 1.33)
			Placebo (n = 205)	‒ 1.09 (‒ 1.49 to ‒ 0.69)	‒ 1.34 (‒ 1.73 to ‒ 0.95)	‒ 1.57 (‒ 2.00 to ‒ 1.14)	‒ 1.68 (‒ 2.11 to ‒ 1.25)
EMPOWUR^a^ [32]	Phase III, R, DB, PC, AC 12 weeks	≥ 18 years old, history of OAB, ≥ 8.0 micturitions/day	Vibegron 75 mg OD (n = 526)	‒ 1.8*	‒ 2.0*	‒ 2.7**	NR
			Tolterodine ER 4 mg OD (n = 417)	‒ 1.6	‒ 1.8***	‒ 2.5	NR
			Placebo (n = 520)	‒ 1.3	‒ 1.4	‒ 2.0	NR
Varano et al. 2021^a^ [36]	Subanalysis 12 weeks	EMPOWUR participants ≥ 65 years old	Vibegron 75 mg OD (n = 242)	‒ 1.9*	‒ 2.0*	‒ 2.7**	NR
			Tolterodine ER 4 mg OD (n = 166)	‒ 1.3	‒ 1.8***	‒ 2.1	NR
			Placebo (n = 220)	‒ 1.0	‒ 1.2	‒ 1.7	NR
		EMPOWUR participants ≥ 75 years old	Vibegron 75 mg OD (n = 75)	‒ 2.1***	‒ 2.0*	‒ 2.6**	NR
			Tolterodine ER 4 mg OD (n = 47)	‒ 1.5	‒2.0*	‒ 2.4	NR
			Placebo (n = 57)	‒ 1.2	‒ 0.4	‒ 0.9	NR
Staskin et al. 2022^a^ [34]	Subanalysis 12 weeks	EMPOWUR participants with OAB dry	Vibegron 75 mg OD (n = 123)	‒ 1.8 (‒ 2.3 to ‒ 1.3)	‒ 2.6 (‒ 3.2 to ‒ 2.0)	NR	NR
			Tolterodine ER 4 mg OD (n = 98)	‒ 1.3 (‒ 1.9 to ‒ 0.8)	‒ 2.0 (‒ 2.7 to ‒ 1.3)	NR	NR
			Placebo (n = 115)	‒ 1.0 (‒ 1.5 to ‒ 0.5)	‒ 1.6 (‒ 2.2 to ‒ 0.9)	NR	NR
		EMPOWUR participants with OAB wet	Vibegron 75 mg OD (n = 403)	‒ 2.1 (‒ 2.4 to ‒ 1.9)	‒ 3.0 (‒ 3.3 to ‒ 2.6)	NR	NR
			Tolterodine ER 4 mg OD (n = 319)	‒ 2.0 (‒ 2.2 to ‒ 1.7)	‒ 2.8 (‒ 3.2 to ‒ 2.4)	NR	NR
			Placebo (n = 403)	‒ 1.7 (‒ 1.9 to ‒ 1.5)	‒ 2.4 (‒ 2.7 to ‒ 2.0)	NR	NR
Newman et al. 2023^a^ [35]	Subanalysis 12 weeks	Women-EMPOWUR participants	Vibegron 75 mg OD (n = 463)	‒ 1.9	‒ 2.1	‒ 2.8	NR
			Tolterodine ER 4 mg OD (n = 364)	‒ 1.7	‒ 1.8	‒ 2.5	NR
			Placebo (n = 459)	‒ 1.4	‒ 1.4	‒ 1.9	NR
EMPOWUR extension^a^ [37]	Phase III, R, DB, AC 52 weeks	EMPOWUR completers	Vibegron 75 mg OD (n = 176)	‒ 2.4 (‒ 2.9 to ‒ 2.0)	‒ 2.2 (‒ 2.5 to ‒ 1.9)***	‒ 3.4 (‒ 4.0 to ‒ 2.7)	‒ 2.5 (‒ 2.8 to ‒ 2.2)***
			Tolterodine ER 4 mg OD (n = 136)	‒ 2.0 (‒ 2.5 to ‒ 1.5)	‒ 1.7 (‒ 2.0 to ‒1.3)	‒ 3.2 (‒ 4.0 to ‒ 2.5)	‒ 1.9 (‒ 2.3 to ‒ 1.6)
COURAGE [38]	Phase III, R, DB, PC 24 weeks^b^	Men ≥ 45 years, OAB symptoms despite pharmacologic BPH treatment	Vibegron 75 mg OD (n = 538)	‒ 2.04 (0.109)*	‒ 2.19 (0.210)**	– 2.88 (0.164)*	NR
			Placebo (n = 542)	‒ 1.30 (0.109)	‒ 1.39 (0.202)	– 1.93 (0.164)	NR

*AC* active-controlled, *BID* twice daily, *BPH* benign prostatic hyperplasia, *CFB* change from baseline, *CI* confidence interval, *DB* double-blind, *ER* extended-release, *LSM* least-squares mean, *NC* not controlled, *NR* not reported, *OAB* overactive bladder, *OD* once daily, *OL* open-label, *PC* placebo-controlled, *R* randomised, *SE* standard error, *UUI* urge urinary incontinence

^a^EMPOWUR was not powered to detect differences between vibegron and tolterodine; the statistical analysis for efficacy compared active drug (vibegron or tolterodine) with placebo only

^b^Outcomes are reported at 12 weeks as LSM (SE)

^*^p < 0.001 vs placebo. **p < 0.01 vs placebo. ***p < 0.05 vs placebo

### Phase III studies

Two multicentre, phase III trials conducted in Japan assessed the efficacy and safety of vibegron in patients with OAB [30, 31]. These trials demonstrated improvements in OAB symptoms, including urinary frequency, urgency episodes, UUI and nocturia (Table 2), with the differences significant versus placebo in the placebo-controlled trial (p < 0.05) [30]. Vibegron also increased voided volume per micturition compared with placebo, with notable efficacy evident from week 4 of treatment. Improvements in HRQOL were observed across several domains, and the treatment was well tolerated [30, 31].

EMPOWUR was a multicentre, international, randomised, double-blind, placebo- and active-controlled phase III study designed to assess the efficacy and safety of vibegron 75 mg once daily in adults with OAB [32]. Participants were randomised in a 5:5:4 ratio to receive vibegron, placebo or extended-release tolterodine 4 mg. The study enrolled 1,518 patients, 90.4% of whom completed the 12-week treatment period. The study population consisted primarily of women (85.2%) and had a mean age of 61 years, while 43% of participants were aged ≥ 65 years. At baseline, participants averaged 11.5 micturitions and 8.1 urgency episodes per day.

At week 12, vibegron demonstrated statistically significant reductions in daily micturitions and UUI episodes compared with placebo (Table 2); vibegron produced a numerically greater magnitude of effect than tolterodine [32]. For urinary frequency, the least-squares (LS) mean change from baseline (CFB) with vibegron was − 1.8 episodes per day, compared with − 1.3 episodes per day with placebo, yielding an LS mean difference of − 0.5 (95% confidence interval [CI] − 0.8 to − 0.2; p < 0.001). Tolterodine demonstrated an LS mean CFB of − 1.6 episodes per day, with an LS mean difference from placebo of − 0.3 (p = 0.10). For UUI, vibegron reduced episodes by − 2.0 per day, compared with − 1.4 per day for placebo, resulting in an LS mean difference of − 0.6 (95% CI − 0.9 to − 0.3; p < 0.0001). Significant reductions in both endpoints were observed with vibegron as early as week 2 and were sustained throughout the study. Efficacy was consistent across subgroups, including treatment-naïve patients and those with prior pharmacotherapy for OAB. It is important to note that EMPOWUR was not powered to detect differences between vibegron and tolterodine; therefore, any comparisons between the two are considered descriptive. The statistical analysis for efficacy compared active drug (vibegron or tolterodine) with placebo only.

Key secondary endpoints in EMPOWUR also demonstrated the superiority of vibegron over placebo [32]. At week 12, the LS mean CFB in urgency episodes was − 2.7 episodes per day with vibegron, compared with − 2.0 episodes per day for placebo, yielding an LS mean difference of − 0.7 (95% CI − 1.1 to − 0.2; p = 0.002). In addition, vibegron significantly increased voided volume per micturition, with an LS mean increase of 23.5 mL compared with 2.2 mL for placebo (LS mean difference 21.2 mL; 95% CI 14.3 to 28.1; p < 0.0001). In participants with OAB classified as wet (OAB wet), 52.4% of those treated with vibegron achieved a ≥ 75% reduction in UUI episodes at week 12, compared with 36.8% in the placebo group (p < 0.0001).

Vibegron was well tolerated in EMPOWUR, with an AE-related discontinuation rate of 1.7%, compared with 1.1% for placebo and 3.3% for tolterodine at week 16 (including the 12-week treatment period and the 4-week follow-up period) [32]. The incidence of hypertension, reported in 1.7% of vibegron-treated participants, was similar to placebo (Table 3). Other AEs, including headache, diarrhoea and nasopharyngitis, were mild and infrequent and occurred at incidences similar to those observed with placebo. Notably, dry mouth, a common AE of antimuscarinic therapies, occurred in 1.7% of vibegron-treated patients, 0.9% of the placebo group and 6.5% of the tolterodine group.

**Table 3:** Safety outcomes with vibegron in significant clinical trials and subgroup analyses

Study, reference	Design follow-up duration, weeks	Population	Treatment	AEs, %	Drug-related AEs, %	Serious AEs, %	AEs leading to discontinuation, %	Hypertension, %
Yoshida et al. 2018 [30]	Phase III, R, DB, PC, AC 12 weeks	≥ 18 years old, history of OAB, ≥ 8.0 micturitions/day	Vibegron 50 mg OD (n = 370)	28.1	7.6	0.3	0.8	0
			Vibegron 100 mg OD (n = 368)	30.4	5.4	0.3	0.8	0
			Imidafenacin 0.1 mg BID (n = 117)	33.3	10.3	0.9	0.9	1.7
			Placebo (n = 369)	27.4	5.1	0.8	0.5	0
Yoshida et al. 2018 [31]	Phase III, OL, NC 52 weeks	≥ 20 years old, history of OAB, ≥ 8.0 micturitions/day	Vibegron 50 mg OD, dose retained (n = 118)	57.8	18.1	NR	NR	NR
			Vibegron 100 mg OD, dose escalated (n = 51)	49.0	11.8	NR	NR	NR
EMPOWUR [32]	Phase III, R, DB, PC, AC 12 weeks	≥ 18 years old, history of OAB, ≥ 8.0 micturitions/day	Vibegron 75 mg OD (n = 526)	38.7	NR	1.5	1.7	1.7
			Tolterodine ER 4 mg OD (n = 417)	38.6	NR	2.3	3.3	2.6
			Placebo (n = 520)	33.3	NR	1.1	1.1	1.7
Varano et al. 2021 [36]	Subanalysis 12 weeks	EMPOWUR participants ≥ 65 years old	Vibegron 75 mg OD (n = 242)	44.7	NR	NR	4.1	1.2
			Tolterodine ER 4 mg OD (n = 166)	42.7	NR	NR	1.6	2.9
			Placebo (n = 220)	37.3	NR	NR	2.1	3.1
		EMPOWUR participants ≥ 75 years old	Vibegron 75 mg OD (n = 75)	49.3	NR	NR	6.3	1.3
			Tolterodine ER 4 mg OD (n = 47)	50.0	NR	NR	4.0	2.1
			Placebo (n = 57)	40.0	NR	NR	0	3.3
Newman et al. 2023 [35]	Subanalysis 12 weeks	Women-EMPOWUR participants	Vibegron 75 mg OD (n = 463)	39.3	13.4	1.3	1.5	1.9
			Tolterodine ER 4 mg OD (n = 364)	39.6	15.7	2.2	3.8	2.7
			Placebo (n = 459)	34.9	11.1	0.9	1.1	1.7
EMPOWUR extension [37]	Phase III, R, DB, AC 52 weeks	EMPOWUR completers	Vibegron 75 mg OD (n = 176)	62.6	NR	3.3	1.5	8.8
			Tolterodine ER 4 mg OD (n = 136)	54.3	NR	4.3	3.4	8.6
COURAGE [38]	Phase III, R, DB, PC 24 weeks^a^	Men ≥ 45 years, OAB symptoms despite pharmacologic BPH treatment	Vibegron 75 mg OD (n = 538)	45.0	9.9	4.3	2.9	9
			Placebo (n = 542)	39.0	9.4	2.9	2.7	8.3

*AC* active-controlled, *AE* adverse event, *BID* twice daily, *BPH* benign prostatic hyperplasia, *DB* double-blind, *ER* extended-release, *NC* not controlled, *NR* not reported, *OAB* overactive bladder, *OD* once daily, *OL* open-label, *PC* placebo-controlled, *R* randomised

^a^Outcomes are reported at 12 weeks as LSM (SE)

Subsequently, an analysis conducted by Frankel and colleagues aimed to determine the clinical meaningfulness of symptom reductions observed in the EMPOWUR trial [33]. Using an anchor-based methodology, the study linked changes in clinical endpoints, such as daily micturitions, urgency episodes and UUI episodes, with patient-reported outcomes, specifically the Patient Global Impression of Change (PGI-C). The analysis identified clinically meaningful thresholds, including a ≥ 15% reduction in micturitions, ≥ 50% reduction in urgency episodes and ≥ 75% and ≥ 90% reductions in UUI episodes, based on patient-perceived improvement.

Results demonstrated that significantly more patients treated with vibegron achieved meaningful symptom reductions compared with placebo [33]. For instance, 56.3% of vibegron-treated patients achieved a ≥ 15% reduction in micturitions compared with 44.6% of placebo recipients (p < 0.001), while 49.3% and 35.2% of vibegron-treated patients experienced ≥ 75% and ≥ 90% reductions in UUI episodes, respectively, compared with 32.8% and 23.5% with placebo (p < 0.0001 and p < 0.001, respectively).

#### Vibegron in OAB with and without incontinence

A subgroup analysis of the EMPOWUR trial assessed the efficacy of vibegron 75 mg in patients with OAB classified as either dry (OAB dry) or OAB wet (Table 2) [34]. Of the 1463 patients in the full analysis set, 336 (23%) were classified as having OAB dry and 1127 (77%) as having OAB wet; notably, a higher proportion of patients with OAB dry were men compared with the overall population (29.5% vs 14.8%), while patients with OAB wet were predominantly women (89.5% vs 70.5%).

At week 12, vibegron demonstrated significant reductions in daily urgency episodes compared with placebo in both subgroups [34]. Similarly, reductions in daily micturitions were significant with vibegron in both subgroups (OAB dry: LS mean difference − 0.8, 95% CI − 1.5 to − 0.1; OAB wet: − 0.5, 95% CI − 0.8 to − 0.1). Improvements were observed as early as week 2 for urgency episodes and week 4 for micturitions and were sustained throughout the 12-week study period. No significant differences were observed between tolterodine and placebo in either subgroup for these endpoints. Responder analyses further highlighted the efficacy of vibegron, including in patients with OAB dry, as 36.9% of those treated with vibegron achieved a ≥ 50% reduction in urgency episodes by week 12 compared with 23.1% with placebo (p < 0.05). The findings were consistent with those in the overall population and underscore vibegron’s ability to address the core symptoms of OAB, regardless of the presence or absence of UUI.

#### Vibegron in women

A prespecified subgroup analysis of the EMPOWUR trial examined the efficacy and safety of vibegron 75 mg in women, who constituted 84.9% (n = 1,286) of the trial population (Table 2) [35]. Women had a mean age of 59.5 years and most (80.9%) were classified as having OAB wet.

At week 12, vibegron demonstrated statistically significant improvements across key efficacy endpoints in women compared with placebo [35]. Reductions in daily micturitions were greater with vibegron (− 1.9) than with placebo (− 1.4; LS mean difference − 0.5, 95% CI − 0.8 to − 0.2). For UUI episodes, vibegron resulted in a mean reduction of − 2.1 compared with − 1.4 with placebo (LS mean difference − 0.7, 95% CI − 1.0 to − 0.4). These improvements were consistent with the overall trial population and highlight vibegron’s efficacy in addressing key OAB symptoms in women. Vibegron also improved HRQOL measures in women, including OAB-q subscale scores for coping, concern, sleep, symptom bother and overall HRQOL (all p < 0.01), as well as in PGI scores for severity, control, frequency, leakage and change (all p < 0.01).

Safety findings revealed a similar incidence of treatment-emergent adverse events (TEAEs) between vibegron (39.3%) and placebo (34.9%), with headache being the most frequently reported TEAE in the vibegron group (4.3% vs 2.6% with placebo; Table 3) [35]. The AE-related discontinuation rate was 1.5% for vibegron, 1.1% for placebo and 3.8% for tolterodine. These data reinforce vibegron’s favourable benefit-risk profile for women with OAB.

#### Vibegron in older patients

An analysis of the EMPOWUR trial evaluated the efficacy and safety of vibegron 75 mg in patients aged ≥ 65 years (n = 628) and ≥ 75 years (n = 179; Table 2) [36].

At week 12, patients treated with vibegron demonstrated significant improvements in OAB symptoms compared with placebo [36]. Among patients aged ≥ 65 years, LS mean reductions from baseline in daily micturitions and UUI episodes were − 1.9 (p < 0.0001) and − 2.0 (p < 0.001), respectively. Similarly, patients aged ≥ 75 years showed LS mean reductions of − 1.7 in micturitions (p < 0.05) and − 2.0 in UUI episodes (p < 0.0001). These changes were consistent with those observed in the overall population (Table 2).

Safety outcomes were favourable, with comparable AE rates for vibegron, placebo and tolterodine (Table 3) [36]. In patients aged ≥ 65 years, common AEs with vibegron included headache, dry mouth and upper respiratory tract infections, while hypertension occurred in 1.2%, which was lower than placebo (3.1%) and tolterodine (2.9%). Among those aged ≥ 75 years, urinary tract infection and diarrhoea were the most frequently reported AEs with vibegron, but cardiovascular-associated AEs were rare and similar to placebo. These findings underscore vibegron’s suitability for older patients, particularly given its minimal risk of anticholinergic-related side effects and drug-drug interactions.

#### Vibegron in long-term therapy

The long-term extension study of the EMPOWUR trial, conducted over 40 weeks with a subsequent 4-week safety follow-up, evaluated the safety, tolerability and efficacy of vibegron 75 mg compared with tolterodine 4 mg extended-release in adults with OAB [37]. Patients who completed the initial 12 week EMPOWUR study continued their original double-blind treatment or, if previously assigned to placebo, were directly randomised to either vibegron or tolterodine. This extension study enrolled 506 participants, with 505 receiving ≥ 1 dose of study medication and 430 completing the study. The primary endpoint was safety, while secondary endpoints included changes in the average daily number of micturitions, urgency episodes and UUI episodes [37].

The long-term safety profile of vibegron was consistent with the 12-week results [32]. The incidence of treatment-emergent AEs was similar between vibegron (62.6%) and tolterodine (54.3%; Table 3). Hypertension was the most common AE reported, occurring in 8.8% and 8.6% of patients, respectively. Dry mouth was more prevalent with tolterodine (5.2%) than vibegron (1.8%). Serious adverse events (SAEs) were rare, and discontinuations due to AEs were less common with vibegron (1.5%) than tolterodine (3.4%). No clinically meaningful changes in laboratory parameters, vital signs or other safety assessments were observed.

The study demonstrated that vibegron provided durable efficacy across all evaluated endpoints. Based on the analysis of the intention-to-treat population, LS mean reductions from baseline to week 52 in micturitions, UUI episodes urgency episodes and total incontinence episodes were greater for vibegron than tolterodine. Specifically, vibegron achieved reductions of − 2.4 micturitions and − 2.2 UUI episodes per day compared with − 2.0 and − 1.7, respectively, for tolterodine. Among patients with OAB wet, 61.0% of those receiving vibegron experienced a ≥ 75% reduction in UUI episodes by week 52, and 40.8% achieved complete resolution of UUI episodes. The efficacy observed in the initial 12-week trial was sustained throughout the 40-week extension, including among patients who switched from placebo to vibegron [37]. These findings reinforce vibegron’s potential as a well-tolerated and effective long-term treatment option for OAB [37].

A phase III open-label, non-controlled study conducted in Japan evaluated the 52-week efficacy and safety of vibegron 50 mg (n = 118) or 100 mg (n = 51) in patients with OAB [31]. Significant improvements from baseline in OAB symptoms, including urinary frequency, urgency episodes, UUI and nocturia were noted at week 4 and maintained at week 52 (Table 2). Improvements in HRQOL were observed and a dose increase to 100 mg improved OAB symptoms without increasing AEs in those patients who did not respond adequately to vibegron 50 mg. The incidence of AEs was 57.8% in 50 mg recipients and 49.0% in 100 mg recipients (Table 3). No novel, clinically significant AEs were seen with long-term treatment.

#### Vibegron in men with concomitant benign prostatic hyperplasia

COURAGE was a phase III, randomised, double-blind, placebo-controlled study designed to evaluate the efficacy and safety of vibegron 75 mg once daily in men aged ≥ 45 years who had persistent symptoms of OAB despite receiving pharmacological treatment for benign prostatic hyperplasia (BPH; Table 2) [38]. The trial included men with OAB symptoms such as ≥ 8 micturitions and ≥ 3 urgency episodes per day over a period of ≥ 2 months, with BPH managed by α-blockers, with or without 5α-reductase inhibitors. Participants were randomised in a 1:1 ratio to receive either vibegron 75 mg or placebo over 24 weeks as an add-on to ongoing treatment with α-blockers, with or without 5α-reductase inhibitors [38]. The coprimary efficacy endpoints of the trial were the changes from baseline to week 12 in the mean daily number of micturitions and urgency episodes.

Results showed that vibegron significantly reduced daily micturition (LS mean difference − 0.74; p < 0.0001) and urgency episodes (− 0.95; p < 0.0001) compared with placebo. Significant improvements were also observed in nocturia (LS mean difference − 0.22; p = 0.002), UUI episodes (− 0.80; p = 0.003), International Prostate Symptom Score (IPSS) storage scores (− 0.9; p < 0.0001) and voided volume per micturition (15.07 mL; p < 0.0001). Efficacy was evident as early as week 2 and maintained throughout the study [38].

Safety data indicated that vibegron was well tolerated, with treatment-emergent AEs occurring in 45.0% and 39.0% of patients in the vibegron and placebo groups, respectively (Table 3). Common treatment-emergent AEs included hypertension, COVID-19, urinary tract infection and haematuria, with no clinically meaningful differences between groups. SAEs were infrequent, and no treatment-related SAEs were reported. Importantly, rates of urinary retention and increases in residual urine volume were comparable between the two groups, and vibegron did not result in significant changes in blood pressure, even among participants with preexisting hypertension [38].

The COURAGE trial concluded that vibegron 75 mg provides significant and clinically meaningful improvements in OAB symptoms among men with pharmacologically treated BPH, with a safety profile consistent with previous studies of vibegron in OAB populations [38].

### Cardiovascular safety of vibegron

A dedicated 28-day, randomised, double-blind, placebo-controlled study evaluated the effects of vibegron 75 mg on ambulatory blood pressure (BP) and heart rate (HR) in patients with OAB [39]. The study randomised 214 adults aged 40–75 years, stratified by age, sex and hypertension status to receive vibegron or placebo. The primary endpoint was the change from baseline to day 28 in mean daytime systolic BP (SBP), with secondary endpoints including changes in diastolic BP (DBP), HR and mean 24 h BP and HR.

Results demonstrated no statistically significant or clinically meaningful differences between vibegron and placebo in changes from baseline for all measured cardiovascular parameters [39]. The LS mean difference for mean daytime SBP was 0.8 mmHg (95% CI − 0.9 to 2.5), and for mean daytime DBP, it was 0.0 mmHg (95% CI − 1.2 to 1.1). Mean daytime HR differed by 0.9 beats per minute (95% CI − 0.3 to 2.0) between groups. Similar findings were observed for 24-h ambulatory measurements, reinforcing that vibegron had no significant pressor effects. No statistically significant between-group difference in the incidence of AEs was noted between vibegron (46.2%) and placebo (25.0%), with hypertension reported in 4.7% and 3.7% of patients, respectively. Notably, no AEs of hypertension with vibegron were considered treatment-related, and one hypertension event was attributed to a prohibited concomitant medication. SAEs occurred in one patient per group (vibegron = postoperative pain; placebo = hypoglycaemia); neither was deemed related to treatment. No deaths or significant cardiovascular events were reported. The safety profile of vibegron was consistent with findings from previous trials. This study supports the cardiovascular safety of vibegron, showing no significant impact on BP or HR, even in subgroups with preexisting hypertension. These findings underscore vibegron’s suitability as a treatment option for OAB, particularly for patients with cardiovascular risk factors.

### Vibegron vs mirabegron

The efficacy and safety of vibegron and mirabegron have been directly compared in at least two clinical trials [40, 41]. A randomised controlled study by Kinjo and colleagues in 199 treatment-naïve postmenopausal women with OAB found that both treatments (at 50 mg/day) provided significant improvements in OAB symptom scores (OABSS), voiding diary parameters and HRQOL measures over 12 weeks, with no significant differences between the two treatments [41]. Rates of TEAEs were comparable, with constipation being the most frequently reported event in both groups.

A multicentre, prospective, randomised, open crossover study by Wada and colleagues further examined these drugs in a cohort of 83 women with OAB, comparing their effects over two consecutive 8-week treatment periods [40]. Both treatments (at 50 mg/day) led to significant improvements in OABSS, nocturia and voided volume, but vibegron resulted in a significantly greater reduction in daytime urinary frequency compared with mirabegron (− 1.5 vs − 0.9 episodes/day; p = 0.016). In addition, a higher proportion of patients expressed a preference for vibegron over mirabegron (53% vs 27%), with the remaining 20% showing no preference. Safety outcomes were generally similar, though an increase in post-void residual volume exceeding 100 mL was observed in two vibegron-treated patients but not in those receiving mirabegron. This difference may not be clinically relevant as it was observed in only two out of 40 patients. The difference was not statistically significant (p = 0.16) and the two patients were elderly (73 and 77 years old) with a baseline PVR of 30 mL and 35 mL, respectively, suggesting possible preexisting voiding dysfunction.

In addition, the real-world adherence and persistence with vibegron compared with mirabegron and anticholinergic therapies for OAB have been evaluated in a retrospective claims analysis [42]. The analysis included data from 4921 patients receiving vibegron, matched with 9842 patients on mirabegron and 9352 on anticholinergics, based on propensity scores that accounted for demographic, clinical and prescription-related variables. The study evaluated adherence, defined as the proportion of days covered (PDC) over the follow-up period, and persistence, defined as the number of days to discontinuation or end of follow-up. Patients treated with vibegron demonstrated significantly higher adherence compared with both mirabegron (mean PDC: 0.67 vs 0.64, p < 0.001) and anticholinergics (0.67 vs 0.58, p < 0.001). In addition, a larger proportion of vibegron-treated patients achieved adherence (PDC ≥ 0.80) compared with mirabegron (49.0% vs 45.1%, p < 0.001) and anticholinergics (49.1% vs 38.5%, p < 0.001). Persistence was also significantly greater with vibegron (median duration of 171 days) compared with mirabegron (128 days; p < 0.001) and anticholinergics (91 days; p < 0.001). Interestingly, among patients discontinuing vibegron, approximately 54% initiated another OAB medication, with nearly half reinitiating vibegron. These findings suggest that vibegron may address common barriers to long-term adherence and persistence in OAB management, such as tolerability and perceived efficacy.

Despite these findings, further research is needed to clarify the long-term comparative benefits of vibegron and mirabegron, particularly regarding treatment adherence, persistence and tolerability in diverse patient populations. As noted in a letter to the editor by Dai and Deng, the absence of a placebo arm in these trials limits the ability to fully assess the relative efficacy of each drug, and future studies should incorporate placebo-controlled designs while also addressing patient comorbidities, including psychological factors that may influence treatment outcomes [43].

## Discussion

The findings from clinical trials and real-world studies demonstrate that vibegron addresses critical unmet needs in OAB management, particularly for patients who are intolerant to or inadequately managed by antimuscarinic therapies. Its favourable safety profile, including minimal cardiovascular effects, distinguishes it from traditional anticholinergics. Vibegron’s high β_3_AR selectivity ensures effective symptom relief with reduced off-target effects, while its once-daily dosing and tolerability may contribute to better long-term adherence and persistence compared with existing therapies.

The ongoing phase IV study, COMPOSUR, is designed to provide real-world insights into the use of vibegron for the treatment of OAB [44]. This prospective, observational study will span 12 months, with an optional extension to 24 months, and includes patients initiating a new course of vibegron. By assessing treatment satisfaction, safety, adherence and persistence, COMPOSUR seeks to complement findings from randomised controlled trials. The primary endpoint focuses on treatment satisfaction using the OAB-SAT-q questionnaire [44].

Vibegron has also shown promise in the treatment of neurogenic bladder. Although clinical data in this population remain limited, promising efficacy has been reported in a paediatric patient with anticholinergic-resistant neurogenic detrusor overactivity [45], as well as in combination therapy in nine paediatric patients with neurogenic bladder [46]. Additionally, an ongoing phase II/III trial is assessing the safety, efficacy and pharmacokinetics of vibegron in children with neurogenic bladder overactivity (NCT05491525).

## Conclusions

Vibegron represents a significant advancement in the pharmacological management of OAB, offering robust efficacy, a favourable safety profile, and potential to improve adherence and persistence compared with traditional therapies.

## Data Availability

No datasets were generated or analysed during the current study.
