# Correction to: Vibegron in overactive bladder: a comprehensive review of efficacy, safety and patient-reported outcomes

**DOI:** 10.1007/s00345-025-06010-8

**Published:** 2025-11-03

**Authors:** Benoit Peyronnet, Benjamin M. Brucker, Cosimo De Nunzio, Christian Gratzke, John Heesakkers, Martin C. Michel, Maurizio Serati, David Staskin, Christopher Chapple

**Affiliations:** 1 Department of Urology, Hopital Pontchaillou, University Hospital of Rennes, Service dUrologie2 Rue Henri Le Guilloux, 35000 Rennes, France; 2 Department of Urology and Obstetrics & Gynaecology, NYU Langone Health, New York, NY USA; 3 Department of Urology, Ospedale Sant’Andrea, Sapienza University of Rome, Rome, Italy; 4 Department of Urology, Faculty of Medicine, Freiburg ImBreisgau, University Freiburg, Baden-Württemberg, Germany; 5 Department of Urology, Maastricht UMC+, Maastricht, Netherlands; 6 Department of Pharmacology, University Medical Center, Johannes Gutenberg University, Mainz, Germany; 7 Department of Obstetrics and Gynecology, Urogynecology Unit, University of Insubria, Varese, Italy; 8 Tufts University School of Medicine, Boston, MA USA; 9 Sheffield Teaching Hospitals, The University of Sheffield, South Yorkshire, Sheffield, UK

**Correction to: World Journal of Urology 43:514 (2025)** 10.1007/s00345-025-05799-8

In the original version of the article, Fig. 1 has been incorrectly published.

The In correct and correct figures are given below

Incorrect figure:
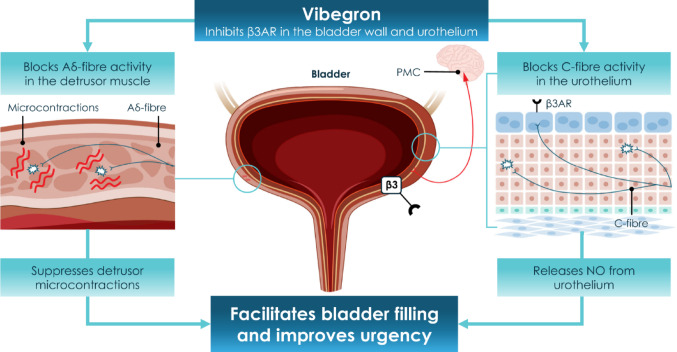


Correct figure:
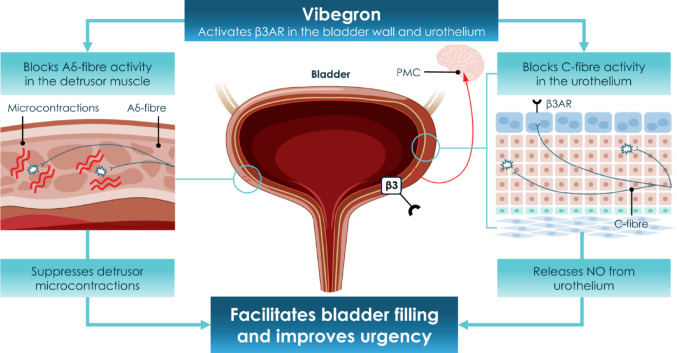


The original article has been corrected.

